# Immunosuppressive Activity of *Artemisia argyi* Extract and Isolated Compounds

**DOI:** 10.3389/fphar.2020.00402

**Published:** 2020-04-08

**Authors:** Amy M. Zimmermann-Klemd, Jakob K. Reinhardt, Anna Morath, Wolfgang W. Schamel, Peter Steinberger, Judith Leitner, Roman Huber, Matthias Hamburger, Carsten Gründemann

**Affiliations:** ^1^Center for Complementary Medicine, Institute for Infection Prevention and Hospital Epidemiology, Faculty of Medicine, University of Freiburg, Freiburg, Germany; ^2^Pharmaceutical Biology, Pharmacenter, University of Basel, Basel, Switzerland; ^3^Signalling Research Centres BIOSS and CIBSS, University of Freiburg, Freiburg, Germany; ^4^Faculty of Biology, University of Freiburg, Freiburg, Germany; ^5^Spemann Graduate School of Biology and Medicine, University of Freiburg, Freiburg, Germany; ^6^Center for Chronic Immunodeficiency, Medical Center Freiburg and Faculty of Medicine, University of Freiburg, Freiburg, Germany; ^7^Center for Pathophysiology, Infectiology, and Immunology, Institute of Immunology, Medical University of Vienna, Vienna, Austria; ^8^Translational Complementary Medicine, Department of Pharmaceutical Sciences, University of Basel, Basel, Switzerland

**Keywords:** Artemisia, immunosuppression, interleukin-2, T cell signalling, sesquiterpene lactones

## Abstract

The need for novel drugs for the treatment of autoimmune diseases is high, since available pharmaceuticals often have substantial side effects and limited efficacy. Natural products are a good starting point in the development of immunosuppressive leads. Since enhanced T cell proliferation is a common feature of autoimmune diseases, we investigated the T cell proliferation inhibitory potential of an extract library of plants used in traditional Chinese medicine. Using a newly established cell-based screening platform, an ethyl acetate extract of *Artemisia argyi* H.Lév. & Vaniot (Asteraceae, *A. argyi*) was found to suppress the proliferation of human primary T lymphocytes *in vitro* in an IL-2-dependent manner. Flow cytometry- and ELISA-based techniques further demonstrated that the *A. argyi* extract reduced the activation and function of T cells. Transcription factor analysis and flow cytometric calcium influx investigations indicated that the immunomodulatory effect was based on specific modification of T cell signaling in a non-cytotoxic manner which is mediated *via* the NFAT pathway and a non-sequestrant inhibition of the calcium influx. A series of guaianolide and *seco*-guaianolide sesquiterpene lactones, as well as a flavonoid, were identified in a previous study as the bioactive compounds in the *A. argyi* extract. The effects of these bioactive compounds were compared to those of the crude extract. The tested sesquiterpene lactones act *via* the transcription factor NFAT and NF-κB, thereby exhibiting their immunosuppressive potential, but have an overall effect on T cell biology on a more-downstream level than the crude *A. argyi* extract.

## Introduction

T cells play a major role in the immune system. A complex mechanism of antigen recognition and signal transduction ensures a highly specific and highly efficient clearance of pathogens.

Upon T cell activation, several adaptor molecules and signaling proteins are phosphorylated to initiate three main axis of signal transduction in T cells. In this process, phosphatidylinositol-4,5-bisphosphate (PIP_2_) is hydrolyzed to generate inositol-1,4,5-trisphosphate (IP_3_) and diacylglycerol (DAG). While DAG is membrane associated, IP_3_ binds to the IP_3_ receptor in the membrane of the endoplasmic reticulum (ER) leading to calcium ER store depletion. Upon calcium ER store depletion, release-activated channels (CRAC channels) mediate a strong store-operated calcium entry (SOCE) (Hogan et al., 2010). The rising calcium concentration in the cytosol causes dephosphorylation and thereby unmasking of the nuclear location sequence of the nuclear factor of activated T cells (NFAT) *via* second messenger and phosphatase activation (Srikanth et al., 2017). On a further axis the mitogen-activated protein kinase (MAPK) pathway is triggered, resulting the formation of activator protein 1 (AP-1) and its nuclear transport (Myers et al., 2019). The third axis induces phosphorylation of nuclear factor of the kappa-light-polypeptide-gene enhancer in B cells inhibitor (IκB), leading to its degradation. Subsequently, the nuclear factor kappa-light-chain enhancer of activated B cells (NF-κB) is released for nuclear transport (Sun, 2012). The transcription factors NFAT, NF-κB, and AP-1 all bind to the *interleukin-2* (*il-2)* gene to allow transcription and secretion of interleukin-2 (IL-2) (Myers et al., 2019). IL-2 autocrinally stimulates T cell proliferation, and thus, is crucial for a proper immune response. Consequently, IL-2 can be linked to immune overreactions.

Overreaction of the immune system can be linked to autoimmune diseases such as rheumatoid arthritis or multiple sclerosis (Chaplin, 2010; Wang et al., 2015). The treatment of autoimmune diseases usually involves different classes of immunosuppressive drugs (Her and Kavanaugh, 2016). Glucocorticoids inhibit the function of immune cells as the activated glucocorticoid receptor directly interferes with the transcription factors NF-κB and AP-1 (van der Laan and Meijer, 2008; Frenkel et al., 2015; Wang et al., 2017). Glucocorticoids are quite effective, but this potency is accompanied by a range of side effects (Ramamoorthy and Cidlowski, 2016). Drugs such as cyclophosphamide or mycophenolate interfere with the cell cycle and, thereby, inhibit lymphocyte proliferation (Allison, 2000; Wang et al., 2015). Despite their clinical efficacy, these drugs also show severe side effects (Allison, 2000; Wang et al., 2015). Biopharmaceuticals, also called biologics, are widely used due to minor toxicity and high levels of specificity. They intervene strongly in the immune system and, therefore, lead to an increased susceptibility to infections and paradoxical inflammation (Her and Kavanaugh, 2016; Moroncini et al., 2017; Wagner, 2019). Small-molecule drugs (e.g., cyclosporine A, tacrolimus, or tofacitinib) interfering in T cell signaling lead to a suppression of T cell proliferation by addressing different molecular targets (Allison, 2000; Tedesco and Haragsim, 2012; Wiseman, 2016), and they all show adverse effects, such as nephrotoxicity and an increased susceptibility to infections (Allison, 2000).

Hence, compounds with novel modes of action and fewer side effects are needed. Natural products remain a promising source for the discovery and development of new drugs. A recent analysis emphasized their relevance by demonstrating that one third of new chemical entities (NCEs) approved by the Food and Drug Administration (FDA) between 1981 and 2014 were based on natural products (Newman and Cragg, 2016). Plant secondary metabolites possess high structural diversity which has likely evolved for serving different biological functions (Atanasov et al., 2015).

Aiming the discovery of new plant derived drugs, we recently tested a library of 435 extracts from plants used in traditional Chinese medicine (TCM), whereby immunosuppressive activity and inhibition of T lymphocyte proliferation *in vitro*, without apparent cytotoxicity, was targeted. One of the best hits in this library was an ethyl acetate extract of *Artemisia argyi* H. Lév. & Vaniot (Asteraceae). *A. argyi* (also called “Chinese mugwort”) grows in China, Japan, and Korea and is traditionally used for the treatment of abdominal pain, dysmenorrhea, uterine hemorrhage, and inflammation (Yun et al., 2016). In previous studies, fatty acids, essential amino acids, sesquiterpene lactones, coumarins, sterols, terpenes, and polyphenols were the main compound classes isolated from *A. argyi* (Bao et al., 2013; Kim et al., 2015). *A. argyi* was recently shown to have anti-inflammatory properties (Yun et al., 2016). The anti-inflammatory effects were supported by *in vivo* experiments that showed reduced cytokine levels and immune infiltration in mouse models for contact dermatitis (Yun et al., 2016) and allergic asthma (Shin et al., 2017). The anti-inflammatory properties of *A. argyi* were linked to some compounds in the extracts, such as the flavonoids jaceosidin, eupatilin, and luteolin, and to a sesquiterpene dimer. These compounds were recently shown to decrease the production of inflammatory mediators and cytokines (Zeng et al., 2014; Li et al., 2018).

We previously showed that the *A. argyi* extract inhibited the proliferation of stimulated human T lymphocytes *in vitro*, and a series of related guaianolides and *seco*-guaianolides was found to be responsible for most of the inhibitory effects of the extract (Reinhardt et al., 2019). In the present study, we aimed to further substantiate the rationale for the use of *A. argyi* as an anti-inflammatory herbal drug. We here address the effects of *A. argyi* extract and selected compounds on the activation and function of T cells *in vitro*, as well as the effects of these compounds on relevant signaling pathways.

## Materials and Methods

### Ethics Approval Statement

Written informed consent was obtained from patients prior to blood donation for research purposes. All experiments conducted on human material were approved by the Ethics Committee of the University of Freiburg (55/14; 11.02.2014). All performed methods are compliant with the regulations of the Ethics Committee of the University of Freiburg.

### Preparation and Cultivation of Human Peripheral Lymphocytes

Preparation and cultivation of human peripheral lymphocytes were performed as indicated (Zimmermann-Klemd et al., 2019). Briefly, peripheral blood mononuclear cells (PBMCs) were isolated from the blood of healthy adult donors, which was obtained from a blood transfusion center (University Medical Center, Freiburg, Germany). Venous blood was centrifuged on a LymphoPrep™ gradient (1.077 g/cm^3^, 20 min, 500 × g, 20°C; Progen, Heidelberg, Germany). After centrifugation cells were washed twice with phosphate buffered saline (PBS) and subsequently cultured in Roswell Park Memorial Institute medium (RPMI) 1640 medium supplemented with 10% heat-inactivated fetal calf serum, 2 mM l-glutamine, 100 U/ml penicillin, and 100 U/ml streptomycin (all from Life Technologies, Paisley, UK). Cells were cultured at 37°C in a humidified incubator with a 5% CO_2_/95% air atmosphere.

### Activation and Treatment of Lymphocytes

Lymphocytes were activated with anti-CD3 (clone OKT3) and anti-CD28 (clone 28.2) mAbs (each 100 ng/ml; both from eBioscience, Frankfurt, Germany) in the presence of medium; cyclosporine A (CsA; 4.16 µM; Sandimmun 50 mg/ml, Novartis Pharma, Basel, Switzerland); camptothecin (CPT; 300 µM: Tocris, Bristol, UK); 0.5% Triton-X 100; plant extract; or isolated compounds from *A. argyi*, as described previously (Zimmermann-Klemd et al., 2019). After cultivation, the cells were used in biological tests.

### Determination of Apoptosis and Necrosis of T Cells

Determination of apoptosis and necrosis of T cells was performed as described previously (Zimmermann-Klemd et al., 2019). Cells were treated for 48 h. Cultured cells were washed with PBS and stained with Annexin V-FITC using the apoptosis detection kit (eBioscience, Frankfurt, Germany) according to instructions of the manufacturer. Propidium iodide (PI; eBioscience, Frankfurt, Germany) was added, and cells were stained for 15 min at room temperature in the dark. The proportion of apoptotic/necrotic lymphocytes was determined by flow cytometric analysis (FACSCalibur instrument; BD Biosciences, Franklin Lakes, NJ).

### Determination of T Cell Proliferation

The proliferation of T lymphocytes was determined using carboxyfluorescein diacetate succinimidyl ester (CFSE) staining, as described previously (Parish et al., 2009; Gründemann et al., 2012). Lymphocytes were isolated, washed twice in cold PBS, and resuspended in PBS at a concentration of 5 × 10^6^ cells/ml. Cells were stained for 10 min at 37°C with CFSE (5 µM; Sigma-Aldrich, St. Louis, MO). The staining reaction was stopped by washing twice with a complete medium. Stained cells were treated for 72 h. The progress of cell division was determined by flow cytometric analysis (FACSCalibur instrument; BD Biosciences, Franklin Lakes, NJ).

### Analysis of Activation Marker of T Cells

The activation state of T lymphocytes was determined *via* cell-surface analysis of CD25 and CD69, as previously reported (Gründemann et al., 2014). Briefly, cells were treated for 24 h. Then, cells were washed with PBS and stained with PE-labeled, anti-CD25 mAbs; FITC-labeled, anti-CD69; and, for the differentiation of CD4^+^ and CD4^–^ T cells, with APC-labeled, anti-CD4 mAbs (all from eBioscience, Frankfurt, Germany) for 20 min at 4°C. Afterward, cells were washed twice with PBS, resuspended, and transferred into FACS vials. The expression of CD25 and CD69 was measured for CD4^+^ and CD4^–^ T cells, respectively, by flow cytometric analysis (FACSCalibur instrument; BD Biosciences, Franklin Lakes, NJ).

### Determination of Cytokine Secretion

After 20 h of treatment, cells were restimulated with PMA (50 ng/ml; Sigma-Aldrich, Taufkirchen, Deutschland) and ionomycin (500 ng/ml; Sigma-Aldrich, Taufkirchen, Deutschland) for 4 h. Supernatants were stored at –20°C. The amount of cytokines was quantified using ELISA technique according to manufacturer's instructions (Affymetrix, Frankfurt, Germany).

### Determination of Cytokine-Producing Cells

Cells were treated for 20 h, as described in section 2.3, and restimulated with PMA (50 ng/ml; Sigma-Aldrich, Taufkirchen, Deutschland) and ionomycin (500 ng/ml; Sigma-Aldrich, Taufkirchen, Germany) for an additional 4 h at 37°C. During this time, they were additionally treated with GolgiPlug (0.5 µl; BD Biosciences, Heidelberg, Germany). After incubation, cells of each sample were divided into three approaches: one for the determination of IL-2, one for the determination of TNFα, one for the determination of IFN-γ. The cells were fixed with 100 µl of 4% PFA (Morphisto, Frankfurt, Germany) for 10 min at room temperature and washed with PBS. Afterward, permeabilization was performed using 100 µl of 1× BD Perm/Wash Puffer (BD Biosciences, Heidelberg, Germany) per sample for 15 min at 4°C. Finally, cells were stained with 1 µl anti-IL-2 or anti-IFN-γ (both Affymetrix, Frankfurt, Germany) for 30 min at 4°C. After two washing steps samples were analyzed by flow cytometric analysis (FACS LSR Fortessa Instrument; BD Biosciences, Franklin Lakes, NJ).

### Analysis of T Cell Degranulation

A CD107a surface staining was performed, as described previously (Gründemann et al., 2013), to determine the T cell degranulation capacity. Cells were treated for 20 h and then restimulated for 4 h with PMA (50 ng/ml; Sigma-Aldrich, Taufkirchen, Deutschland) and ionomycin (500 ng/ml; Sigma-Aldrich, Taufkirchen, Deutschland). To each well containing 200 µl of cell suspension, 2.5 µl (~0.25 µg) of PE-conjugated, anti-CD107a mAbs (eBioscience, Frankfurt, Germany) was added. After incubation at 37°C for 1 h, 2 µl of 1/10 diluted GolgiStop (Becton Dickinson, Franklin Lakes, NJ) was added per well, and the cells were incubated for another 3 h. Samples were analyzed by flow cytometric analysis (FACSCalibur instrument; BD Biosciences, Franklin Lakes, NJ).

### Reporter Cell Experiments for the Determination of NFAT-, NF-κB-, and AP-1 Activity

A 96 well F-bottom cell culture plate was coated with anti-human CD3 mAb (clone OKT3, 1 µg/ml, 50 µl/well) or PBS (unstim. control) at 4°C over-night. Reporter cells (Jutz et al., 2016) were harvested, washed twice with PBS, and seeded in a 5% FCS RPMI cell culture medium (0.15 × 10^6^ cells in 200 µl/well). Cells were treated with inhibitors (1 nM SP100030 for AP-1, 5 µg/ml cyclosporine A for NFAT, and 20 µM parthenolide for NF-κB), plant extract or isolated compounds from *A. argyi* or remained untreated (unstim. control, stim. control). Cells were incubated at 37°C for 8 h (AP-1) or 24 h (NFAT and NF-κB). Cells were washed twice with PBS and the expression of eGFP was determined by flow cytometric analysis (FACSCalibur instrument; BD Biosciences, Franklin Lakes, NJ).

### Determination of Intracellular Calcium

Jurkat cells (0.5 × 10^6^) were stained in 200 µl RPMI medium, containing 1% FCS, 2.6 µM Fluo3 AM (Life Technologies, Carlsbad, California), 5.5 µM FuraRed AM (Invitrogen, Carlsbad, California), and 0.1% (w/v) Pluronic F-127 (Invitrogen, Carlsbad, California) in the presence of test substances (30 µg/ml *A. argyi* extract or 10 µg/ml compound/compound mix; merely for the determination of the calcium ER store depletion) for 45 min at 37°C. For differentiation of calcium ER store depletion and SOCE, the staining solution was further supplemented with 0.6 mM ethylenediaminetetraacetic acid (EDTA). Cell suspensions were gently mixed every 10 min. After staining cells were resuspended in 100 µl of 1% FCS RPMI medium containing the test substance (30 µg/ml *A. argyi* extract or 10 µg/ml compound/compound mix; merely for the determination of the calcium ER store depletion). For the measurement of calcium influx, 50 µl of cell suspension were prewarmed (37°C, 5 min) in 700 µl of 1% FCS RPMI medium supplemented with 0.6 mM EDTA (for differentiation of ER store depletion and SOCE) and the test substance (30 µg/ml *A. argyi* extract or 10 µg/ml compound/compound mix; merely for the determination of the calcium ER store depletion). Baseline calcium levels were determined by flow cytometric measurement (FACS CyAn ADP; Beckman Coulter, Brea, California) for 1 min. Afterward, calcium influx was induced *via* stimulation with anti-CD3 mAbs (clone OKT3, 1 µg/ml). After 2 min, the test substance (30 µg/ml *A. argyi* extract or 10 µg/ml compound/compound mix) was added, followed, 30 s later, by the addition of calcium dichloride (1 mM). Calcium influx was measured for 5 min.

### Analysis of Data

For statistical analysis, data were processed with Microsoft Excel and SPSS software (Version 22.0, IBM, Armonk, USA). Statistical significance was determined with the SPSS software by a one-way ANOVA followed by Dunnett's *post hoc* pairwise comparisons. Values are presented as mean ± standard deviation (SD) for the indicated number of independent experiments. The asterisks represent significant differences from controls (*p < .05).

### Tested Compounds

The compounds used in this work were isolated from *A. argyi* ethyl acetate extract in a previous study (Reinhardt et al., 2019).

## Results

### Anti-Proliferative Effects of the *A. argyi* Extract on T Lymphocytes

**Figure 1:**
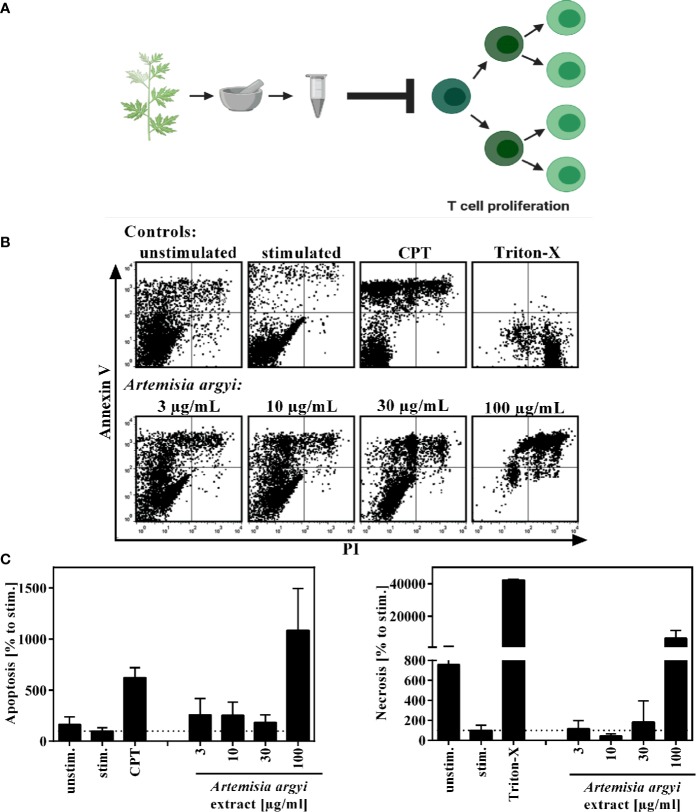
Overview of recently published results **(A)** and effect of the *A. argyi* extract on the viability of T lymphocytes **(B, C)**. Expanded human lymphocytes (2 × 10^5^) were left unstimulated (unstim.) or were stimulated (stim.) with anti-CD3 and anti-CD28 mAbs (100 ng each) and incubated for 48 h with medium (unstim., stim.), camptothecin (CPT; 300 μM), Triton-X 100 (0.5%) or the *A. argyi* extract. Annexin V-FITC and PI double stainings were performed after incubation. The proportions of viable, necrotic, and apoptotic cells were determined by flow cytometry. Data are depicted as dot plots **(B)**, and the proportion of apoptotic/necrotic lymphocytes in relation to the stimulated control was determined from two independent experiments. Data are depicted as mean ± SD **(C)**.

Previously, we evaluated the effect of 435 plant extracts from a focused extract library of TCM plants on the proliferative capacity of primary expanded human T lymphocytes *in vitro*. The ethyl acetate extract of *A. argyi* inhibited the proliferation of human T lymphocytes in a concentration-dependent manner, with a half maximal inhibitory concentration (IC_50_) of 16.2 µg/ml (Reinhardt et al., 2019) (**Figure 1A**). The focused library also comprised extracts from other plants of the Asteraceae family (**Supplement 2**), but none of these exhibited notable activity in the assay.

To verify that the observed immunosuppressive activity was not due to cytotoxicity of the extract, flow cytometry analysis was performed to analyze the apoptosis and necrosis events of the cells. Using Annexin V/PI double staining, we found that the *A. argyi* extract had no toxic effects in the concentration range that was used for the mechanistic studies (**Figures 1B, C**).

### Effects of the *A. argyi* Extract on T Lymphocyte Function

**Figure 2:**
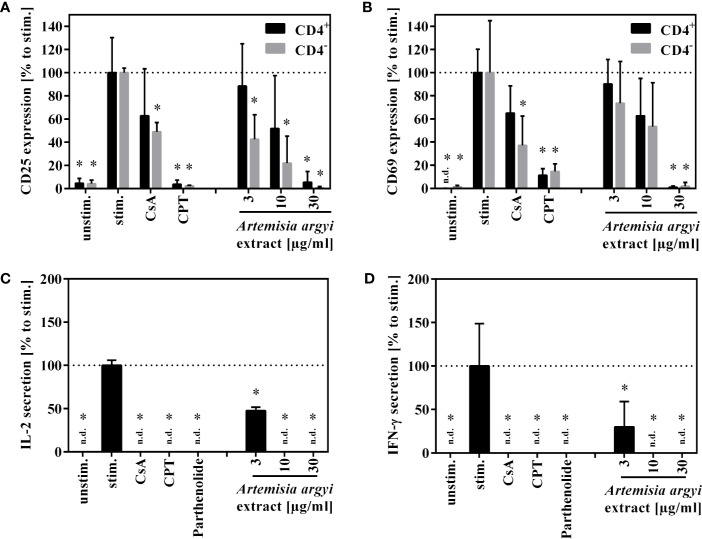
Effects of *A. argyi* extract on the function of T lymphocytes. For **(A**, **B)**, lymphocytes (2 × 10^5^) were left unstimulated (unstim.) or were activated with anti-CD3 and anti-CD28 mAbs (100 ng each and incubated for 24 h with medium (unstim., stim.), cyclosporine A (CsA; 4.16 µM), camptothecin (CPT; 300 µM), or the *A. argyi* extract. Cells were stained with anti-CD69-FITC and anti-CD4-APC **(A)** or anti-CD25-PE and anti-CD4-APC **(B)**. Expression of surface markers (CD69, CD25, CD4) was analyzed by flow cytometry, and the amounts of activated CD4^+^ and CD4^–^ T lymphocytes were determined. For **(C**, **D)** expanded lymphocytes (2 × 10^5^) were incubated for 20 h with medium (unstim., stim.), cyclosporine A (CsA; 4.16 µM), camptothecin (CPT; 300 µM), parthenolide (20 µM) or the *A. argyi* extract. Subsequently, the cells were stimulated with PMA/Ionomycin for 4 h at 37°C. IL-2 **(C)** and IFN-γ **(D)** were quantified in the supernatants by ELISA. The results of three different experiments are summarized and are depicted as mean ± SD in relation to the untreated, stimulated control. *p < 0.05. n.d.= below detection limit.

Next, the effect of the *A. argyi* extract on the effector function of human T cells was examined. Stimulation of T lymphocytes *via* the T cell receptor (TCR) leads to the expression of the activation markers CD25 and CD69. We found that after TCR stimulation the treatment of T cells with the *A. argyi* extract significantly and concentration-dependently lowered CD25 and CD69 expressions (**Figures 2A, B**). This was the case in both the CD4^+^ and CD4^–^ T cells. In addition, we observed a significantly reduced CD25 expression of CD4^–^ T cells after treatments with low concentrations (3 and 10 µg/ml) of the *A. argyi* extract (**Figure 2A**).

Upon activation, T cells secrete IL-2, which is important for proliferation, and interferon-γ (IFN-γ), which defines T cell function. The *A. argyi* extract significantly suppressed IL-2 as well as the IFN-γ secretion capacity of activated T lymphocytes (**Figures 2C, D**).

### Influence of the *A. argyi* Extract on the Transcription Factors AP-1, NFAT, and NF-κB

**Figure 3:**
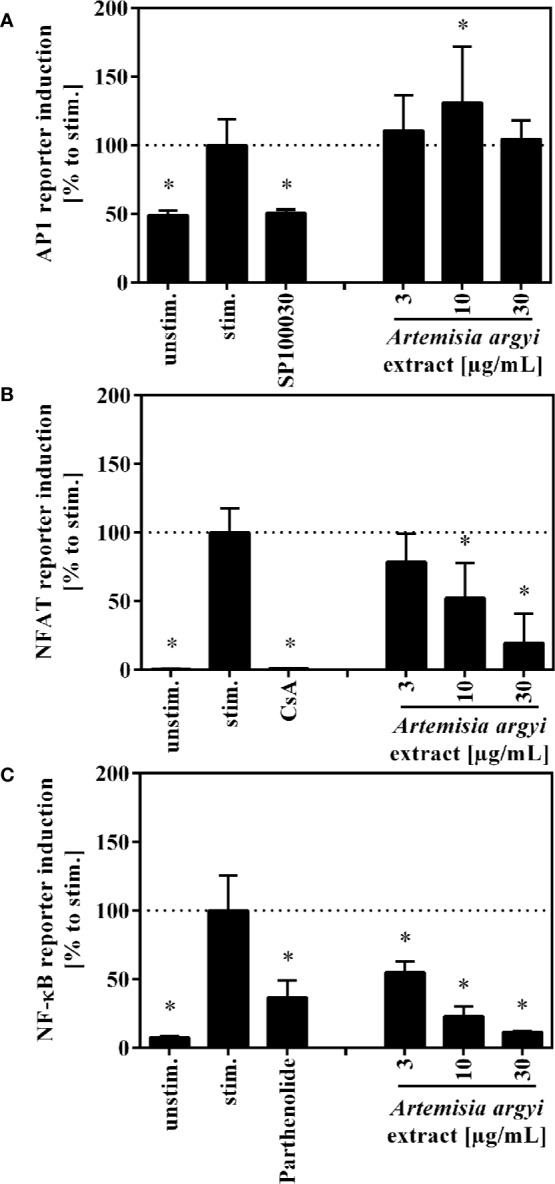
Effect of the *A. argyi* extract on the activity of transcription factors AP-1 **(A)**, NFAT **(B)** and NFκB **(C)** in Jurkat T cells. Jurkat reporter cells (0.15 × 10^6^) were seeded on a cell culture plate, coated with anti-CD3 mAbs, and incubated with medium, inhibitors (AP-1: 1 nM SP100030, NFAT: 4.16 µM CsA, NF-κB: 20 µM parthenolide) or the *A. argyi* extract in different concentrations for 8 (AP-1) or 24 h (NFAT and NF-κB). The GFP expression was measured by flow cytometry after incubation. The results of three independent experiments are summarized and depicted as mean ± SD. *p < 0.05.

To understand how the activation and proliferation of human T cells is inhibited by the *A. argyi* extract, we analyzed the effect of the *A. argyi* extract on the transcription factors AP-1, NFAT, and NF-κB. All three transcription factors regulate the transcription of the *il-2* gene, which is a key regulator in these processes. Jurkat T cell reporter lines (Ratzinger et al., 2014; Jutz et al., 2016), in which eGFP is fused to the response elements of these transcription factors, were used for this purpose. After stimulation with anti-CD3 mAbs, activity of the transcription factors was quantified *via* flow cytometry. The treatment with the *A. argyi* extract did not change the anti-CD3-induced AP-1 activity in comparison to untreated activated AP-1 reporter cells (**Figure 3A**). In contrast, NFAT and NF-κB activity was reduced in a concentration-dependent manner by the *A. argyi* extract (**Figures 3B, C**). In summary, the reporter cell experiments pointed to a specific suppression of NFAT and NF-κB activities, but not to suppression of AP-1.

### Effects of the *A. argyi* Extract on TCR-Induced Calcium Signaling

The previous experiments demonstrated that the *A. argyi* extract lowered activity of NFAT, which was induced by TCR triggering. Influx of calcium ions into the cytosol is upstream regulated of NFAT activation (Robert et al., 2011). Hence, we performed calcium flux experiments to determine whether the calcium influx to the cytosol was impaired. Inhibition of calcium influx would prevent the translocation of NFAT to the nucleus and, consequently suppress NFAT activity.

**Figure 4:**
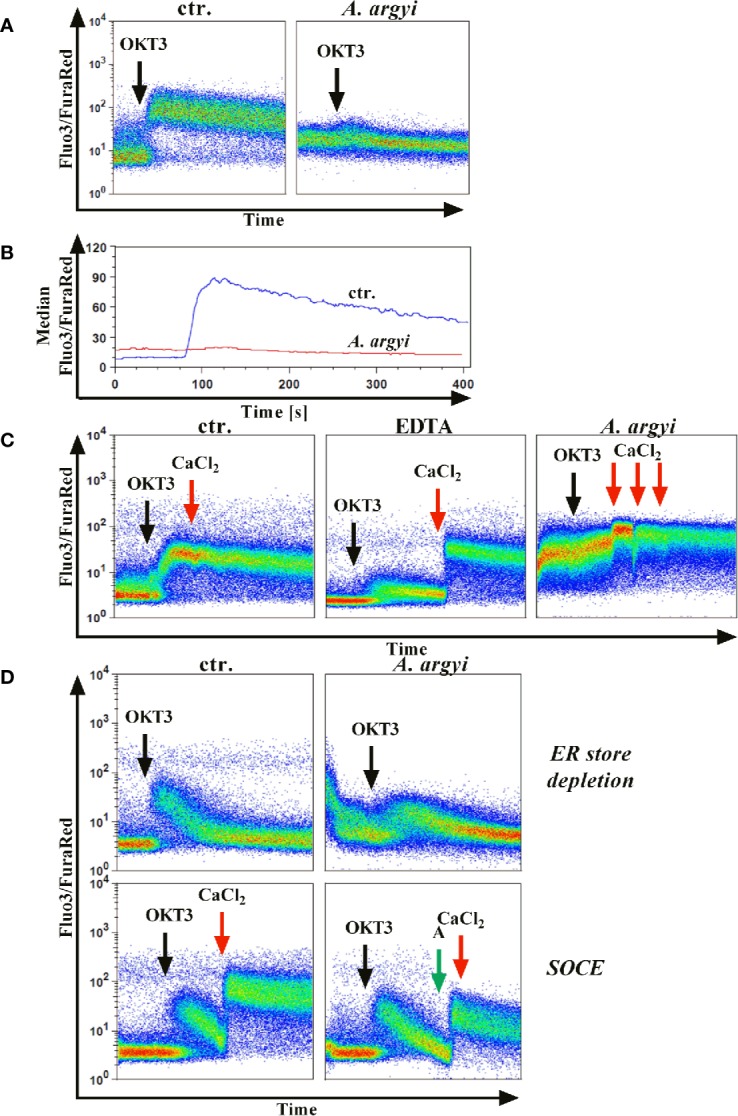
Effect of the *A. argyi* extract on the calcium influx in Jurkat T cells. Jurkat cells (0.5 × 10^6^) were stained with Fluo3 and FuraRed for 45 min at 37°C in the presence of medium, 0.6 mM EDTA or 30 µg/ml *A. argyi* extract. The results show the anti-CD3-induced (black arrows) calcium influx as the Fluo3/FuraRed ratio **(A)** and the median of this ratio **(B)** for untreated cells (ctr.) and cells treated with 30 µg/ml *A. argyi* extract. Calcium influx was induced by anti-CD3 stimulation (black arrows) of the TCR **(C)**, and, after 1 min 1 mM calcium dichloride was added (red arrows). The calcium influx (ratio Fluo3/FuraRed) of untreated cells (ctr.), cells treated with 0.6 mM EDTA, and cells treated with 30 µg/ml *A. argyi* extract are shown. The ER store depletion in presence of the *A. argyi* extract was analyzed **(D)**. Jurkat cells (0.5 × 10^6^) were stained with Fluo3 and FuraRed in a calcium-free medium for 45 min at 37°C. To determine the ER calcium store depletion, 30 µg/ml *A. argyi* extract was present in the medium during staining and measurement, and the ER store depletion was triggered by TCR stimulation (black arrows). The control cells remained untreated. For determination of the SOCE, cells were stained, and the ER calcium store depletion was induced by TCR stimulation (black arrows). After 2 min 30 µg/ml *A. argyi* extract (green arrows) were added and, after another 30 s, 1 mM calcium dichloride (red arrows) was added. For the control (ctr.), 1 mM calcium dichloride was added directly after depletion of the ER calcium store. The results show the calcium influx (the ratio Fluo3/FuraRed) of the untreated cells (ctr.) and the cells treated with 30 µg/ml *A. argyi* extract.

Jurkat cells were stained with the calcium indicators Fluo3 and FuraRed and treated with the *A. argyi* extract. Subsequently, calcium influx was induced by TCR stimulation using an anti-CD3 mAbs. The experiments demonstrated that the treatment of the cells with the *A. argyi* extract led to a complete suppression of the calcium influx (**Figures 4A, B**).

Next, we sought to characterize the inhibition of the calcium influx. Jurkat cells were treated with the *A. argyi* extract or the calcium ion chelator EDTA to determine whether the inhibition was mediated *via* chelation of calcium. As expected, a strong calcium influx was measured for untreated cells (control) after TCR stimulation (**Figure 4C**). This influx was prevented in EDTA-treated cells but restored by the addition of calcium dichloride to the medium (**Figure 4C**). In contrast, in cells treated with the *A. argyi* extract, the calcium influx capability could not be restored with the addition of increasing concentrations of calcium dichloride (**Figure 4C**). This suggested that the *A. argyi* extract did not inhibit the calcium influx through calcium chelating properties.

Upon TCR activation by antigen binding, the opening of a calcium channel in the membrane of the ER initiates calcium release from ER stores (ER store depletion). The depletion of these intracellular calcium stores causes the formation of calcium release-activated channels (CRAC) channels, which in turn leads to a strong influx of calcium from the extracellular space to the cytosol (store-operated calcium entry, SOCE) and allows replenishment of the calcium stores in the ER (Robert et al., 2011). To discriminate between these two options, we stained Jurkat cells with Fluo3 and FuraRed in a medium supplemented with EDTA. During the staining process, the cells were treated with the *A. argyi* extract in a calcium-free setting to ensure a calcium-free, extracellular compartment. Cells were stimulated *via* TCR to induce the calcium influx. Given that the SOCE was prevented, calcium store depletion could be measured. In comparison to the untreated cells (control), treatment with the extract lowered calcium ER store depletion (**Figure 4D**). To determine whether the SOCE was also inhibited by the *A. argyi* extract, or just prevented due to the lacking depletion of ER calcium store, we stained Jurkat cells with Fluo3 and FuraRed in a medium supplemented with EDTA, and induced the calcium ER store depletion by TCR stimulation. Next, the extract and calcium dichloride were added and the SOCE was measured. The medium of control cells was supplemented with calcium dichloride directly after depletion of the intracellular calcium stores. The results showed that the *A. argyi* extract also lowered the SOCE from the extracellular space (**Figure 4D**).

In summary, the strong reduction of calcium influx upon TCR stimulation after treatment with the *A. argyi* extract resulted from suppression of the ER calcium store depletion and a reduction of the SOCE.

### Effects of Isolated *A. argyi* Compounds on the Activation and Function of T Lymphocytes

**Figure 5:**
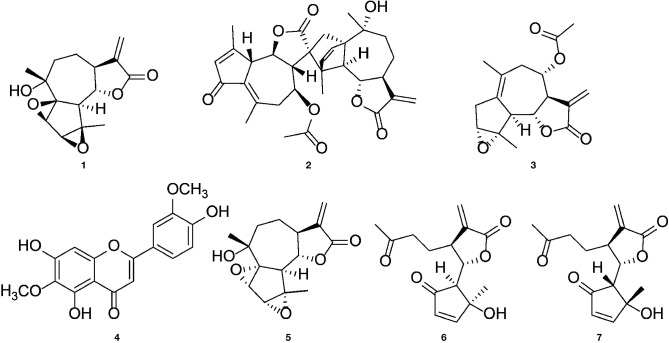
Chemical structures of (1*S*)-artecanin **(1)**, 8-acetyl-artanomaloide **(2)**, arteglasin A **(3)**, jaceosidin **(4)**, (1*R*)-canin **(5)**, (4*S*, 5*S*, 6*S*, 7*S*)- and (4*R*, 5*R*, 6*S*, 7*S*) -*seco*-tanapartholide **(6** and **7)**.

We previously isolated a series of sesquiterpene lactones and flavones from the active extract (**Figure 7A**), some of which showed significant inhibitory effects on T lymphocyte proliferation (Reinhardt et al., 2019) (**Figure 1A**). Several of the most-active compounds were tested: two stereoisomeric guaianolides, artecanin and canin (**1** and **5**), artanomaloide (**2**), arteglasin A (**3**) differing from **1** and **2** in the decoration of the 7-membered ring, the moderately active flavone jaceosidin (**4**), and two stereoisomeric *seco-*guaianolides, *seco*-tanapartholides B and A (**6** and **7**) (**Figure 5**).

To better understand their contribution to the activity of the extract, their influence on activation, cytokine production, and degranulation capacity of human T lymphocytes was investigated.

**Figure 6:**
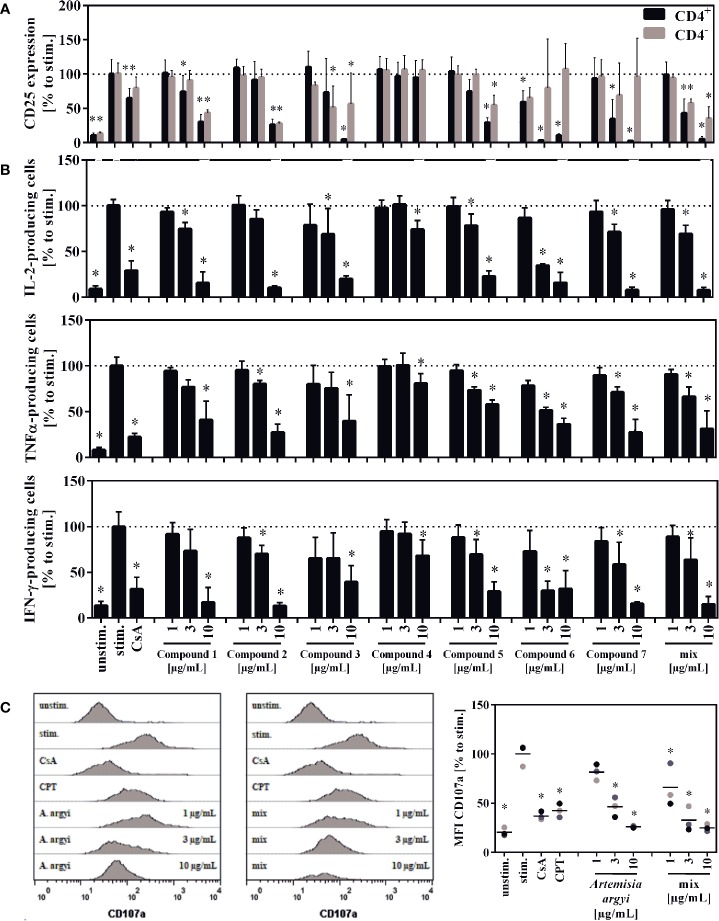
Effect of the isolated compounds and the compound mix on activation **(A)** IL-2-, TNFα-, and IFN-γ-producing T cells **(B)** and the degranulation capacity of T cells **(C)**. For **(A)**, lymphocytes (2 × 10^6^) were stimulated with anti-CD3 and anti-CD28 mAbs (100 ng each) and incubated for 48 h with medium (unstim., stim.), cyclosporine A (CsA; 4.16 µM), the compounds, the compounds mixture, or the *A. argyi* extract **(B)**. Cells were re-stimulated with PMA (500 µg/ml) and ionomycin (500 ng/ml) and treated with GolgiPlug for 4 h. The cells were fixed, permeabilized, and stained with anti-IL-2, anti-TNFα or anti-IFN-γ mAbs. The amount of IL-2-, TNFα-, and IFN-γ-secreting cells was determined *via* flow cytometry. Results from four independent experiments were summarized and are depicted as mean ± SD in relation to the untreated, stimulated control. *p < 0.05. For **(C)** lymphocytes (2 × 10^5^) were stimulated with anti-CD3 and anti-CD28 mAbs (100 ng each). Afterward, cells were incubated for 20 h with medium (unstim., stim.), cyclosporine A (CsA; 4.16 µM), camptothecin (CPT; 300 μM), the compound mixture, or the *A. argyi* extract. Cells (except the unstim. control) were restimulated with PMA (500 µg/ml) and ionomycin (500 ng/ml) for 4 h and stained with anti-CD107a-PE. The amount of degranulating T lymphocytes, as indicated by a CD107a surface expression, was determined *via* flow cytometry. Data are depicted as histogram plots, and data of three independent experiments are summarized as mean ± SD. *p < 0.05.

The compounds **1**, **2**, **3**, **5**, and the compound mix significantly reduced the expression of CD25 in CD4^–^ and CD4^+^ T cells at 10 and/or 3 µg/ml. Compound **6** suppressed the CD25 expression in CD4^+^ T cells at all tested concentration levels, while compound **7** suppressed it at 10 and 3 µg/ml. Compounds **6** and **7** had no significant effect on CD4^–^ T cells. The isolated flavone **4** showed no effect on CD25 expression (**Figure 6A**).

All isolated compounds, with the exception of compound **4**, significantly and strongly suppressed IL-2-producing cells at 10 µg/ml. While the effect was only miniscule for the flavone (**4**), compounds **1**, **3**, **5**-**7**, and the mix also showed significant inhibition at 3 µg/ml. A similar pattern was observed for the suppression of tumor necrosis factor α-(TNFα) and IFN-γ-producing cells. The strongest suppression of IL-2- and IFN-γ-producing cells was observed from compound **6** (**Figure 6B**).

Upon release of perforin and granzymes, T lymphocytes express the lysosomal-associated membrane protein 1 (LAMP-1, CD107a) on their surface. Analysis of the LAMP-1 surface expression, showed concentration-dependent effects for the mixture of all compounds and the *A. argyi* extract (**Figure 6C**).

### Effects of Isolated *A. argyi* Compounds on the Transcription Factors NFAT and NF-κB

**Figure 7:**
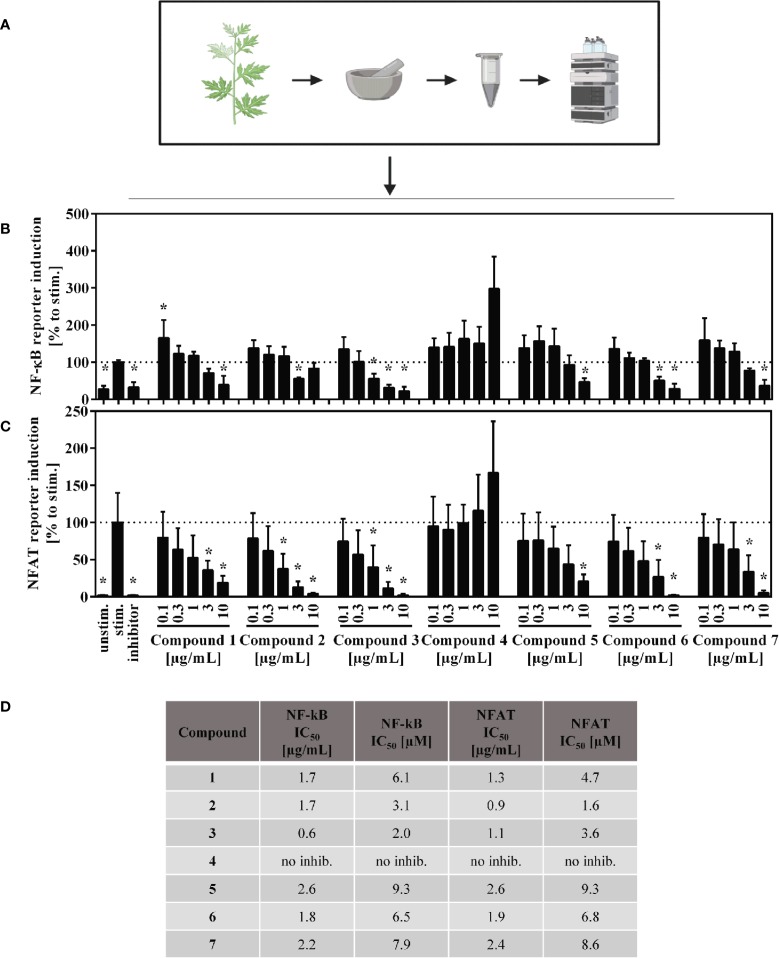
Overview of the published fractionation process **(A)** and effect of the A. argyi compounds on NF-κB **(B)** and NFAT **(C)** activity and corresponding IC_50_ values **(D)** in Jurkart T cells. Jurkat reporter cells (0.15 × 10^6^) were seeded on a culture plate, coated with anti-CD3 mAbs, and incubated with medium, inhibitors (NFAT: 4.16 µM CsA, NF-κB: 20 µM parthenolide) or *A. argyi* compounds in different concentrations for 24 h. The GFP expression was measured by flow cytometry. The results of three independent experiments are summarized and depicted as mean ± SD. *p < 0.05 **(B, C)**. The calculated IC_50_ values are presented in µg/ml and in µM, as stated **(D)**.

Jurkat-based NFAT and NF-κB reporter experiments were performed to shed light on the interaction of compounds by manipulation the T lymphocyte signaling. For the sesquiterpene lactones, but not for the flavone (**4**), a concentration-dependent, suppressive effect on the NFAT pathway was found, with IC_50_ values < 10 µM (< 3 µg/ml) (**Figures 7C, D**). Likewise, NF-κB activity was significantly decreased by all compounds except compound **4** (**Figures 7B, D**). We here focused on the effects of the *A. argyi* extract and isolated compounds on the NFAT signaling pathway.

Thus we tested whether the isolated compounds were able to inhibit TCR-induced calcium signaling. To this end, we looked at both the ER calcium store depletion (**Supplement 1**, left panel) and the SOCE (**Supplement 1**, right panel), using the methodology, described above. Single compounds and a mixture of all isolated compounds were tested with the assumption that the mixture would, at least in part, mimic the activity of the whole extract. The *A. argyi* extract was used as a control. A slight inhibition of calcium influx from the ER combined with a significant time delay was observed for compound **5**. A similar delay was observed for the compound mix and, to a lesser extent, for compounds **1**, **6**, and **7**. Compounds **2** and **3** increased the intensity of the calcium influx. However, neither the single compounds nor the compound mix inhibited the calcium influx as effectively as did the *A. argyi* extract (**Supplement 1**).

## Discussion

We recently found that an ethyl acetate extract of *A. argyi* suppressed the *in vitro* proliferation of human primary T lymphocytes in a concentration-dependent, non-cytotoxic manner (**Figure 1**) and we also isolated a series of compounds that were responsible for this activity (Reinhardt et al., 2019).

Stimulation of TCR promotes the surface expression of the transmembrane C-type lectin CD69 and the alpha-chain of the IL-2 receptor (CD25) on T cells (Malek, 2008). The *A. argyi* extract lowered the expression of both activation markers (**Figures 2A, B**) and of IL-2 production (**Figure 2C**) in stimulated T lymphocytes. As IL-2 is pivotal for lymphocyte proliferation, inhibition of IL-2 production could explain the observed inhibition of T cell proliferation. Further, the IFN-γ secretion was reduced after treatment with the *A. argyi* extract (**Figure 2D**). Our findings provide evidence for IL-2-mediated, anti-inflammatory properties of the extract, likely *via* IL-2-mediated T lymphocyte proliferation inhibition, and thereby corroborate the traditional use of *A. argyi* as an anti-inflammatory herbal drug (Yun et al., 2016).

The IL-2-dependent suppression of T lymphocyte proliferation by the *A. argyi* extract is linked to suppression of the transcription factors NFAT and NF-κB (**Figures 3B, C**), while AP-1 activity was not affected (**Figure 3A**). ER calcium store depletion and the SOCE were inhibited *via* a non-sequestrant mechanism (**Figures 4A–D**). Our observation that it is impossible to restore the calcium influx by generating an overage of calcium dichloride in the outer cell compartment points to an irreversible blockage of the calcium channels. Otherwise, binding and dissociation homeostasis would trigger a stronger calcium influx without binding of the active constituent(s) of the *A. argyi* extract to the calcium channels. The effect of the extract was comparable to that of the calcium chelator EDTA. Inhibition of the ER calcium store depletion and SOCE explain the observed reduced NFAT activity.

To correlate the effects found for the *A. argyi* extract to the compounds from the extract, selected T cell proliferation-inhibiting compounds were investigated analogously. Jaceosidin (**4**) showed no effect on either NFAT or NF-κB reporter cells, which is in accord with its weak inhibition of all three pro-inflammatory cytokines tested. A comparable inhibition of TNFα expression by jaceosidin (**4**) has been reported (Li et al., 2018). Thus, it is unlikely for jaceosidin to contribute significantly to the observed activity of the extract. All tested sesquiterpene lactones were shown to inhibit NFAT and NF-κB binding to the DNA. The inhibition of DNA binding of NF-κB can presumably be compared to the effects published for other sesquiterpene lactones, such as helenalin (a guaianolide) and parthenolide (a germacranolide) (García-Piñeres et al., 2001; García-Piñeres et al., 2004). Parthenolide inhibits the IκB kinase complex β (IKKβ) by alkylating a cysteine residue in its activation loop (Kwok et al., 2001). Less is known about the effect of sesquiterpene lactones on the NFAT pathway, which was the focus of this work. Only helenalin was previously shown to suppress abundance and nuclear translocation of NFATc2 (Berges et al., 2009). The stereoisomeric guaianolides artecanin (**1**) and canin (**5**), as well as the guaianolide dimer **2** and arteglasin A (**3**) showed very similar effects to the *A. argyi* extract in general. However, in the ER store depletion and for the SOCE, the activity of the single compounds was not comparable to that of the extract. Only canin (**5**), but not its stereoisomer artecanin (**1**), showed some inhibition of ER store depletion, which did not affect the SOCE. Thus, canin (**5**) might be part of the explanation for the inhibition of the ER store depletion by the *A. argyi* extract. The specific CD25 inhibition by the two *seco*-guaianolides **6** and **7** in CD4^+^ cells but not inCD4^–^ cells is unusual and deserves further investigation. However, this inhibition is not reflected in the activity of the *A. argyi* extract. Although most of the observed activity from the *A. argyi* extract can be attributed to the presence of the tested guaianolides and *seco*-guaianolides, none of the isolated compounds showed sufficient inhibition of the ER calcium store depletion or the SOCE (**Supplement 1**). This was also true for a mixture of the compounds, suggesting that other, as yet unidentified compounds in the extract are responsible for this activity.

The initial screening (Reinhardt et al., 2019) included extracts from additional herbal drugs of the family Asteraceae, but none of these inhibited T cell proliferation at the concentrations tested. The lack of activity can be possibly explained, by an absence of sesquiterpene lactones from the extracts (*Carthamus tinctorius*, *Artemisia scoparia*, and *Artemisia apiacaea*). So far, sesquiterpene lactones have only been reported from *Artemisia capillaris* (Feng et al., 2017) and *Centipeda minima* (Eisenbrand and Tang, 2011), and their concentrations in the plants were very low, in the range of 0.001% in *A. capillaris* (Wu et al., 2012; Feng et al., 2017). Furthermore, the strength of T cell proliferation inhibition may also depend on the specific sesquiterpene scaffold, as significant differences were observed between eudesmanes, guaianolides, and *seco*-guaianolides (Reinhardt et al., 2019). All reported sesquiterpene lactones from *C. minima* are structurally related to helenalin, which is a known NF-κB inhibitor (Lyss et al., 1997; Eisenbrand and Tang, 2011).

We ascertained different modes of action for the capacity of the *A. argyi* extract to inhibit the proliferation of T cells. We demonstrated that the *A. argyi* extract, as well as different guaianolides isolated from it, inhibited the activity of NFAT and NF-κB. Moreover, the crude extract suppressed the TCR-induced calcium influx, but neither the isolated single compounds nor a mixture of these sesquiterpene lactones showed similar effects. This suggests that the *A. argyi* extract likely contains compounds affecting signaling on a more upstream target than the compounds isolated thus far. Our findings corroborate the notion of a multitarget effect of herbal extracts possibly resulting in pharmacological synergism (Fürst and Zündorf, 2015; Colalto, 2018). The study also demonstrates that the established cell-based screening platform approach is a powerful tool for identifying and characterizing potential immunosuppressive leads from natural product sources.

## Data Availability Statement

The datasets generated for this study are available on request to the corresponding author.

## Ethics Statement

The studies involving human participants were reviewed and approved by Ethics Committee of the University of Freiburg, Engelberger Straße 21, 79106 Freiburg. The patients/participants provided their written informed consent to participate in this study.

## Author Contributions

AZ-K performed the experiments, analyzed the data, prepared the figures, and wrote the draft manuscript. JR prepared **Figure 5** and wrote the draft manuscript regarding results and discussion on "Effects of isolated compounds" and the discussion of other tested Asteraceae. JR, AM, WS, PS, JL, RH, MH, and CG contributed to the design, implementation of the research, and in finalizing the manuscript.

## Funding

Gefördert durch die Deutsche Forschungsgemeinschaft (DFG) im Rahmen der Exzellenzstrategie des Bundes und der Länder – EXC – 2189 – Projektnummer 390939984. (Funded by the Deutsche Forschungsgemeinschaft (DFG, German Research Foundation) under Germany's Excellence strategy – EXC – 2189 – Project ID: 390939984.) Furthermore, this work was financially supported by the Software AG Foundation, DAMUS-DONATA e.V. and PRIAM-based foundation.

## Conflict of Interest

The authors declare that the research was conducted in the absence of any commercial or financial relationships that could be construed as a potential conflict of interest.

## Abbreviations

AP-1, activator protein 1; APC, allophycocyanin; CD, cluster of differentiation; CFSE, carboxyfluorescein succinimidyl ester; CPT, camptothecin; CsA, cyclosporine A; CRAC channel, calcium release activated channel; DAG, diacylglycerine; DMSO, dimethyl sulfoxide; EDTA, ethylenediaminetetraacetic acid; ELISA, enzyme-linked immunosorbent assay; ER, endoplasmic reticulum; FACS, fluorescence-activated cell sorting; FCS, fetal bovine serum; FDA, Food and Drug Administration; FITC, Fluorescein isothiocyanate; GFP, green fluorescent protein; HPLC, High-performance liquid chromatography; IFN-, interferon-; IκB, nuclear factor of kappa light polypeptide gene enhancer in B-cells inhibitor; IL-, interleukin; IC_50_, half maximal inhibitory concentration; IP3, inositol 1,4,5-trisphosphate; mAb, monoclonal antibody; MAPK, mitogen-activated protein kinase; NCEs, new chemical entities; NFAT, Nuclear factor of activated T-cells; NF-κB, nuclear factor kappa-light-chain-enhancer of activated B cells; p, p-value; Par, parthenolide; PIP_2_, phosphatidylinositol 4,5-bisphosphate; PBMC, peripheral blood mononuclear cell; PBS, phosphate buffered saline; PE, phycoerythrin; PI, propidium iodide; PMA, phorbol-12-myristat-13-acetate; RPMI, Roswell Park Memorial Institute medium; SD, standard deviation; SOCE, store-operated calcium entry; TNF-α, tumor necrosis factor alpha; TCM, traditional Chinese medicine; TCR, T cell receptor.
