# Supplementary material for: Immunosuppressive Activity of Artemisia argyi Extract and Isolated Compounds
Source: Front Pharmacol. 2020 Apr 8;11:402. doi: 10.3389/fphar.2020.00402 (PMC7157444; doi:10.3389/fphar.2020.00402)
Supplement: Supplementary file 1 [file DataSheet_1.pdf]

Immunosuppressive activity of *Artemisia argyi* extract and isolated compounds

Amy M. Zimmermann-Klemd[1‡], Jakob K. Reinhardt[2‡], Anna Morath[3,4,5], Wolfgang W. Schamel[3,4,6], Peter Steinberger[7], Judith Leitner[7], Roman Huber[1], Matthias Hamburger[2], Carsten Gründemann[8*]

[1]Center for Complementary Medicine, Institute for Infection Prevention and Hospital Epidemiology, Faculty of Medicine, University of Freiburg, Freiburg, Germany

[2]Pharmaceutical Biology, Pharmacenter, University of Basel, Basel, Switzerland

[3]Signalling Research Centres BIOSS and CIBSS, University of Freiburg, Freiburg, Germany

[4]Faculty of Biology, University of Freiburg, Freiburg, Germany

[5]Spemann Graduate School of Biology and Medicine, University of Freiburg, Freiburg, Germany

[6]Center for Chronic Immunodeficiency, Medical Center Freiburg and Faculty of Medicine, University of Freiburg, Freiburg, Germany

[7]Center for Pathophysiology, Infectiology, and Immunology, Institute of Immunology, Medical University of Vienna, Vienna, Austria

[8]Translational Complementary Medicine, Department of Pharmaceutical Sciences, University of Basel, Basel, Switzerland

[‡] contributed equally to this work

*** Correspondence:**

Prof. Dr. Carsten Gründemann, PhD

carsten.gruendemann@unibas.ch

Keywords: Artemisia, immunosuppression, Interleukin-2, T cell signaling, sesquiterpene lactones

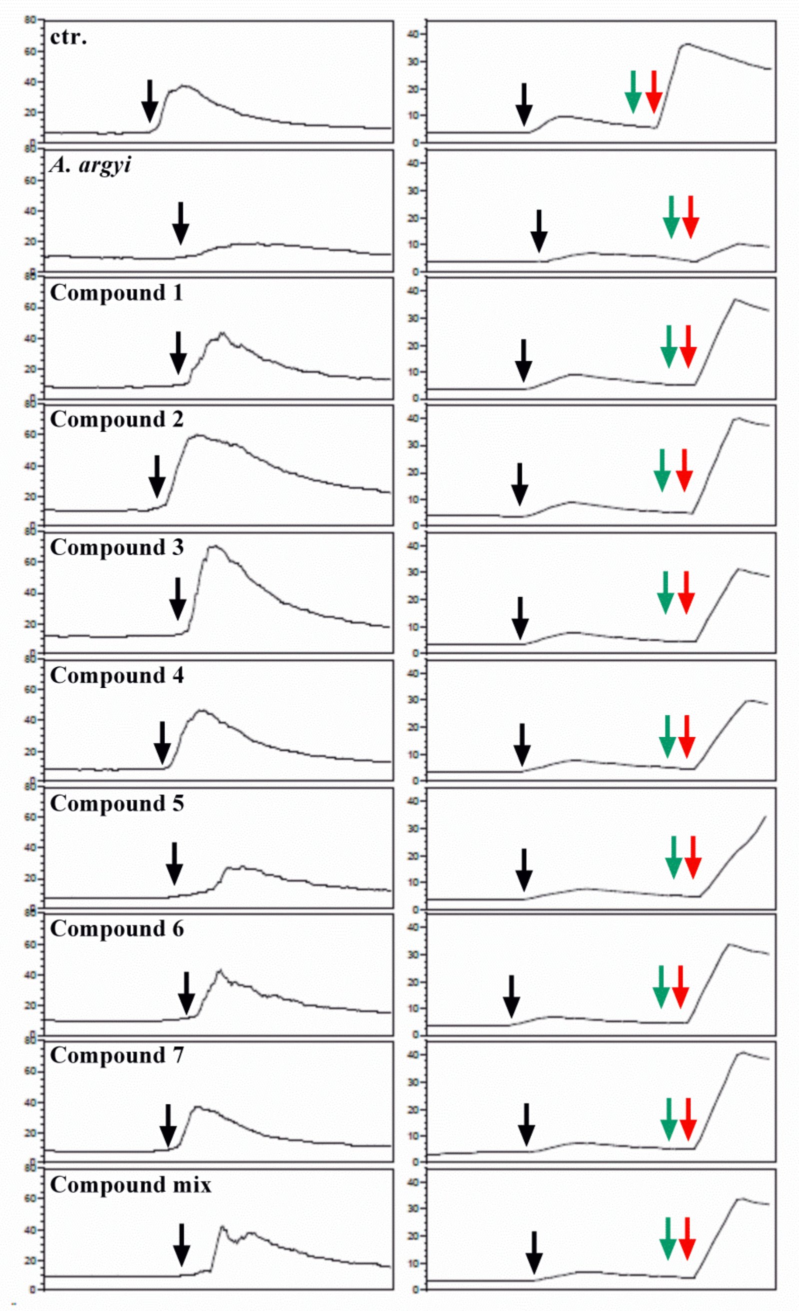

Supplement 1: Effect of isolated compounds and compound mix on the calcium flux in Jurkat T cells, in comparison to *A. argyi* extract. Jurkat cells (0.5 x 10^6) were stained with Fluo3 and FuraRed in calcium-free medium for 45 min at 37°C. During the staining process and measurement 30 µg/mL *A. argyi* extract or 10 µg/mL of the compounds, or 10 µg/mL of compound mix were present in the medium (only left panel). The calcium flux was induced by addition of the anti-CD3 mAbs (black arrows). For the investigation of SOCE, extract, compounds or compound mix were added (green arrows) after ER store depletion and calcium dichloride was supplemented (red arrows) subsequently. The results show the kinetics of calcium flux (median Fluo3/FuraRed) of untreated cells and cells treated with 30 µg/mL *A. argyi* or 10 µg/mL of each compound.

Supplement 2: Table of all tested Asteraceae.

Family	Species	Organ	Extracts
Asteraceae	*Centipeda minima*	Herb	EtOAc ASE
Asteraceae	*Centipeda minima*	Herb	DCM ASE
Asteraceae	*Centipeda minima*	Herb	MeOH ASE
Asteraceae	*Carthamus tinctorius*	Flowers/Buds	EtOAc ASE
Asteraceae	*Carthamus tinctorius*	Flowers/Buds	DCM ASE
Asteraceae	*Carthamus tinctorius*	Flowers/Buds	MeOH ASE
Asteraceae	*Artemisia apiacea*	Herb	EtOAc
Asteraceae	*Artemisia apiacea*	Herb	DCM ASE
Asteraceae	*Artemisia apiacea*	Herb	MeOH ASE
Asteraceae	*Artemisia argyi*	Herb	EtOAc
Asteraceae	*Artemisia argyi*	Herb	DCM ASE
Asteraceae	*Artemisia argyi*	Herb	MeOH ASE
Asteraceae	*Artemisia capillaris*	Herb	EtOAc
Asteraceae	*Artemisia capillaris*	Herb	DCM ASE
Asteraceae	*Artemisia capillaris*	Herb	MeOH ASE
Asteraceae	*Artemisia scoparia*	Herb	EtOAc
Asteraceae	*Artemisia scoparia*	Herb	DCM ASE
Asteraceae	*Artemisia scoparia*	Herb	MeOH ASE
